# A case report of rigidity and recurrent lower limb myoclonus: progressive encephalomyelitis rigidity and myoclonus syndrome, a chameleon

**DOI:** 10.1186/s12883-018-1176-3

**Published:** 2018-10-18

**Authors:** Aurélie Degeneffe, Marie Dagonnier, Alain D’hondt, Jose Antonio Elosegi

**Affiliations:** 1 Department of Neurology, CHU Ambroise Paré Hospital, Boulevard John Fitzgerald Kennedy 2, 7000 Mons, Belgium; 2 Intensive Care Unit, CHU Ambroise Paré Hospital, Mons, Belgium

**Keywords:** PERM, Myoclonus, Rigidity, Stiff person, Stiff man

## Abstract

**Background:**

Progressive encephalomyelitis with rigidity and myoclonus (PERM) syndrome is a rare neurological condition. Its clinical characteristics include axial and limb muscle rigidity, myoclonus, painful spasms and hyperekplexia. Diagnosis of this disease can be very challenging and optimal long-term treatment is unclear.

**Case presentation:**

We report a case of a 62 year old patient admitted for repetitive myoclonus and rigidity in the lower limbs progressing since 10 years, associated with a fluctuating encephalopathy requiring stays in Intensive Care Unit. Multiple diagnostics and treatment were proposed, unsuccessfully, before the diagnosis of PERM syndrome was established. In association with the clinical presentation, a strong positive result for GAD (glutamic acid decarboxylase) antibodies lead to the diagnosis of PERM syndrome.

**Conclusions:**

PERM syndrome is a rare disease and its diagnosis is not easy. Once the diagnosis is established, the correct treatment should follow and could be lifesaving, regardless of a delayed diagnosis. Maintenance of long-term oral corticotherapy is suggested to prevent relapses.

## Background

Progressive encephalomyelitis with rigidity and myoclonus (PERM) syndrome is a rare neurological condition. PERM is suggested to be a more severe variant of the stiff person syndrome (SPS) [1–6]. Its clinical characteristics include muscle rigidity (both axial and limb), myoclonus often stimulus-sensitive and painful spasms [1, 2, 4–9]. This condition is often associated with hyperekplexia due to brainstem dysfunction [3, 10–12]. This brainstem dysfunction is manifesting in alteration of consciousness, cerebellar ataxia and respiratory insufficiency due to breathing and swallowing difficulties [6, 13, 14].

Seizures are often observed [1, 3, 6, 14–21]. This syndrome can present with an insidious onset, as well as an acute or subacute presentation, or exacerbations on a chronic course [3–5, 11, 14, 20]. This condition has previously been associated with the glutamic acid decarboxylase (GAD), dipeptidyl-peptidase-like protein-6 (DPPX) antibodies and glycine receptor (GlyR) serum antibodies [3, 14, 22]. The latter is associated with a disruption of the inhibitory glycinergic synaptic transmission, which is prominent in the spinal cord and brainstem [5, 13]. Abnormal cerebrospinal fluid (CSF) is seen and additional denervation on the electromyography (EMG) [2, 3, 6, 11, 18, 23, 24]. Immunomodulation using corticosteroids, intravenous (IV) immunoglobulin, plasma exchange or cyclophosphamide are described as an effective treatment [1, 24]. Intrathecal Bacflofen has also successfully been applied [1, 2]. Initial diagnosis of PERM is not easy and literature is limited regarding the long-term course of the syndrome. Herein, we report an unusual presentation of PERM in a patient with previous diagnosis of spinal myoclonus, neuroleptic malignant syndrome (NMS) and multiple episodes of relapse.

## Case presentation

The clinical story of this 62 year old woman started in 2007 with lower back pain, lumbar muscular contractures and back rigidity. After being examined by an orthopaedic surgeon, she was diagnosed with congenital scoliosis. A lumbar interbody fusion was subsequently performed with no significant impact on the symptoms.

Between 2007 and 2014, she presented multiple episodes of lower limb myoclonus. Episodes were often associated with hyperthermia. A diagnosis of spinal myoclonus was suggested and a treatment with clonazepam began.

In 2014, she presented with lower limb myoclonus, confusion, hyperthermia and acute respiratory failure. She was admitted to the intensive care unit (ICU). Differential diagnoses at the time included NMS versus meningoencephalitis. Nevertheless, no past history of neuroleptic use was identified and multiple attempts of lumbar puncture were unsuccessful. Large spectrum antibiotics were administered with favourable clinical evolution.

She finally presented in our hospital in our emergency department (ED) in January 2017 for recurrent myoclonus and a sudden onset of bilateral leg weakness in the context of an influenza infection. Upon arrival at the ED, moderate hypotension and fever (39.4 °C) were observed. Neurological examination showed bradyphrenia, however she followed one-step commands. There was a motor deficit of the lower extremities scored 4 on the Medical Research Council scale (MRC) [25], diminished and symmetrical knee and ankle jerks, myoclonus and rigidity in the lower limbs. Initial laboratory tests showed normal white cell count with moderately increased C reactive protein (CRP; 39 mg/l, normal: < 5 mg/l). Creatine kinase (CPK) was elevated at 52144 U/L (normal: 10–170 UI/l) with an acute renal insufficiency (clearance of 14 ml/min). Clonazepam doses were increased from 1 mg to 2 mg three times daily and antibioc therapy with amoxicillin/clavulanic acid was empirically started.

The day after admission in the neurology ward alteration of the level of consciousness, persistent hypotension and respiratory distress were observed and the patient was subsequently admitted to ICU. In the following days, there was a progressive deterioration of the motor function in the lower limbs until paraplegia was reached while lower limb rigidity and myoclonus persisted. Patient contact was lost with respiratory failure, necessitating invasive mechanical ventilation during one week.

Brain and spinal magnetic resonance imaging (MRI) revealed a pituitary adenoma, cervical osteoarthritis and thoraco-lumbar discopathy without stenosis. EMG showed continuous rhythmic motor unit activity within the axial muscles. CPK continued to rise in the following days to peak of 74,410 U/L. CSF analysis was normal, polymerase chain reaction (PCR) for herpes simplex virus 1 (HSV1), HSV 2 and enterovirus were negative (Table 1).

**Table 1:** Diagnostic work-up

Imaging	Brain MRI	Pituitary adenoma
	Spinal MRI	Cervical osteoarthritis and thoraco-lumbar discopathy without stenosis
EMG	Continuous rhythmic motor unit activity within the axial muscles	
EEG	No epileptic activity	
CSF	Normal	
Laboratory tests	CRP	39 mg/L
	CPK	74,410 U/L
	CrCl	14 ml/min
	HIV	Negative
	ANA	Negative
	ANCA	Negative
	Anti-double-stranded DNA AB	Negative
	Antibody to extractable nuclear antigens	Negative
	ASMA	Negative
	AAA	Negative
	Anti-GBM Antibody	Negative
	Paraneoplastic syndromes	Negative
	Complement C3 and C4	Negative
	Mycoplasma	Negative
	Influenza	Negative
	Enterovirus	Negative
	Adenovirus	Negative
	Borrelia Burgdorferi	Negative
	Cytomegalovirus	Negative
	Cardiolipine antibody	Negative
	Lupus anticoagulant	Negative
	Thyroid function	Normal
	Paraneoplastic neurological antibodies (anti-Amphiphysin, anti-Hu, anti-Yo, anti-CV2, anti-Ri, anti-Ma2/Ta, anti-Sox1, anti-Titin)	Negative
	GAD65	95

In doubt, anti-epileptic treatment with levetiracetam (2 g twice daily) was started. Nonetheless, a consecutive twenty-four-hour electroencephalogram (EEG) demonstrated numerous muscular artefacts but no epileptic activity (Table 1).

An extensive blood investigation (Table 1) was performed including: human immunodeficiency virus (HIV), anti-nuclear antibody (ANA), anti-neutrophil cytoplasmic antibody (ANCA), Anti-double-stranded DNA antibody, antibody to extractable nuclear antigens, anti-mitochondrial antibody (AMA), anti-smooth muscle antibody (ASMA), Anti-actin antibody (AAA), Anti-glomerular basement membrane antibody (anti-GBM Ab), paraneoplastic syndromes, complement C3 and C4, mycoplasma, influenza, enterovirus, adenovirus, borrelia burgdorferi, cytomegalovirus, cardiolipine antibody, lupus anticoagulant, thyroid function and paraneoplastic neurological antibodies (anti-Amphiphysin, anti-Hu, anti-Yo, anti-CV2, anti-Ri, anti-Ma2/Ta, anti-Sox1 and anti-Titin). All test results were either normal or negative. However, our patient was tested strongly positive for the antibodies to GAD (95; normal values 0–5). Together with the clinical presentation this ultimately led to the diagnosis of PERM syndrome.

Initial treatment was therefore started with gamma-globulins (0.4 g/kg/day) during 5 days with poor results. Concomitant corticotherapy was started with IV hydrocortisone (50 mg, three times daily) for 10 days, which was then continued orally. The treatment was continued with five sessions of plasma exchange (2 L). Recovery of her consciousness followed within days and myoclonus ceased. She was extubated one week after the end of plasma exchange sessions. Total duration of the ICU stay accounted 33 days. The patient was then transferred to the neurology ward where she recovered progressively with respect to motor function; she was able to walk with a walker when discharged to a rehabilitation centre.

While still in rehabilitation, our patient had a short relapse of the myoclonus in March 2017, only after she was already shortened on her corticosteroids and anti-epileptics. Decision was made to keep her on a regimen of levetiracetam 2 g twice a day and hydrocortisone 20 mg twice daily. She left the rehabilitation centre in May 2017 with a favourable evolution despite persistent mild lower proximal limb weakness (scored 4 on the MRC scale) and gait ataxia.

In August 2017, seven months after admission, she had no relapse of the myoclonus nor the rigidity and was able to walk without assistance. Hydrocortisone was reduced to 10 mg twice daily.

In October, she was again admitted for intermittent episodes of myoclonus (less than 60 min). Myoclonus rapidly disappeared after treatment with IV methylprednisolone (1 g) for three consecutive days. Steroids dose were increased again to 20 mg twice daily. She is now followed monthly at the outpatient clinic and levetiracetam treatment has been progressively stopped while hydrocortisone is maintained at the same dose.

## Discussion and conclusions

PERM is a challenging diagnosis due to several reasons. The condition, firstly described by Whitely et al. in 1976, is relatively rare [7]. Diagnosis is mainly clinical, as (in most cases) there is an absence of signs on imaging. Increased signal intensity of the cervical cord and lower brainstem in T2 MRI sequences was reported in only two cases [8, 26]. EMG and antibodies in serum and CSF could help to confirm the diagnosis. Initial clinical presentation can be very unspecific and uncommon, and this case is no exception. Common differential diagnoses of PERM syndrome are stiff person syndrome, paraneoplastic SPS and NMS [9, 17]. In this case, we can also add spinal myoclonus to the list and potentially scoliosis.

In retrospect, the initial lower back pain and rigidity could have been the premises of the PERM syndrome symptoms. Lumbar spinal fusion was performed in regards to the scoliosis indication. Unfortunately, it had no efficacy on the rigidity symptoms. Moreover, the patient later developed recurrent episodes of lower limb myoclonus. These were, in association with the past history of spinal fusion, wrongly diagnosed as spinal myoclonus.

The episode of myoclonus presented by our patient in 2014, associated with hyperthermia, was considered as an episode of NMS. It was previously reported by Xu et al. that PERM could be mimicking NMS due to its significant overlap in symptoms [17]. In this case, muscle rigidity, fever, autonomic nervous system dysfunction and altered mental status, common to both entities, could have been taken in favour of NMS. Nevertheless, generalised myoclonus, especially its history of recurrent pattern, and even more the lack of evidence of neuroleptic use should have alerted the clinician to intensify the diagnostics.

Elevation of CPK in NMS is reported up to at least four times the upper limit. In the context of PERM, elevation of CPK is less frequently described. Literature is not particularly rich on the topic, but we identified three reported cases with normal CPK values [5, 21, 27], while five described elevated CPK values between 408 IU/l and 4779 IU/l [4, 11, 17, 23, 28].

Besides the clinical presentation, antibodies can be determinative in diagnosing this syndrome. Initially both SPS and PERM syndromes were associated with the GAD antibodies. The most recent literature estimates that there is a 60–80% correlation between GAD positive antibodies and SPS [2, 10, 18, 19, 23, 29, 30]. According to Schmidt et al. GAD positive antibodies would only be present in 25% of the PERM cases [19, 30]. Discovered in association with PERM cases in 2008, GlyR antibodies are absent in most cases of SPS [31]. Unfortunately, GlyR antibodies testing was not available in our institution at the time.

Presumably, GAD antibodies cause a reduced brain GABA, which is consistent with the occurrence of seizures. A four channel EMG study showed defective inhibition and exaggerated excitation of the brainstem reflexes mediated by dysfunction of the inhibitory GABA and glycine neurons [32]. Interestingly, post-mortem analysis of PERM cases shows inflammation of the brainstem [23].

Brainstem involvement can be used to differentiate SPS from PERM. There is a controversy about PERM being part of the spectrum of SPS and considered as SPS-plus version of the main syndrome pain [33]. Initial clinical presentation does indeed show significant overlap in symptoms. Nevertheless, the fluctuating course of our patient with severe relapses definitely supports the diagnosis of PERM.

The presence of GAD antibodies is not specific to PERM syndrome. It is associated with a number of other conditions such as diabetes mellitus, schizophrenia or bipolar disease, Parkinson disease, cerebellar disorders and neuropathic conditions. Patients with type 1 diabetes mellitus usually show GAD65 antibody titers at only less than 20, while patients with neurological autoimmune disorders have serum antibody values greater than 20, usually even greater than 100 [22]. Level of GAD65 antibodies in our patient was increased to 95. However, its association with the clinical features of our case and its evolution leave little doubt to our final diagnosis.

Treatment recommended for PERM patients includes immunomodulation using corticosteroids, IV immunoglobulins, plasma exchange or cyclophosphamide [1, 24]. Intrathecal Bacflofen has also been used successfully [1, 2]. The prognosis after treatment can be fluctuating.

Literature is more limited regarding long-term clinical course of PERM. It has been suggested that the syndrome might stay latent during several years and suddenly decompensate by an infection. Up to 18% of PERM cases are reported with chronic course and acute exacerbations [14, 17]. Recurrence has been reported from a few weeks after disease onset up to nine years [1, 4, 8, 11, 12, 31, 34, 35]. In most cases, relapses are related to corticosteroid discontinuation, as in our case. Often, the dosage of corticosteroids is increased at the time of relapse [8, 11, 22]. However, when there is no satisfactory response, more aggressive immunosuppressive therapy may be required, such as cyclosporine, azathioprine and rituximab [5, 12, 14, 31, 35]. Also other immunotherapies have been described at the time of relapse: plasma exchange and cyclophosphamide [36]. Long-term treatment is described with oral corticosteroids variating from 8 mg/day to 20 mg/day combined with Azathioprine 50 mg/day to 150 mg/day [1, 6, 24, 35]. The combination of oral corticosteroids with Diazepam or Clonazepam and intrathecal Baclofen has also been reported [1, 24].

In the current case nearly 10 years went by, after onset of initial symptoms, before the right diagnosis was found. Cases reporting delayed diagnosis PERM are very rare in the literature. One case was identified with a six month delayed diagnosis of PERM, also with good response to immunoglobulin treatment [17]. After literature review we found three case reports with a delayed diagnosis of SPS, with delays ranging from four months up to three years. The first patient responded well to a treatment combination of diazepam, baclofen, dexamethasone, azathioprine and plasma exchange, the patient maintained moderate rigidity without spasms at a follow-up of five months [37]. The second case was treated by immunoglobulins with good response at a follow-up of two months [38]. The third case received an IV treatment of diazepam, no relapse at six months follow-up [39].

In conclusion, PERM syndrome is a rare disease and its diagnosis is not easy, which is underlined by this case report. Once the diagnosis is established, the correct treatment should follow, which could be lifesaving. Maintenance of long term oral corticotherapy is suggested to prevent relapses. Moreover, it appears that regardless of a delayed diagnosis, a good response may be obtained with adequate treatment in autoimmune central nervous system (CNS) disorders such as PERM. However this report only describes a single case, more data need to be examined in order to generalize these conclusions.

## Abbreviations

AAA: Anti-actin antibody
ANA: Anti-nuclear antibody
ANCA: Anti-neutrophil cytoplasmic antibody
anti-GBM Ab: anti-glomerular basement membrane antibody
CNS: Central nervous system
CPK: Creatine kinase
CSF: Cerebrospinal fluid
DPPX: Dipeptidyl-peptidase-like protein-6
ED: Emergency department
EMG: Electromyography
GAD: Glutamic acid decarboxylase
GlyR: Glycine receptor
HIV: Human immunodeficiency virus
HSV: Herpes simplex virus
ICU: Intensive care unit
IV: Intravenous
MRC: Medical Research Counsil
MRI: Magnetic resonance imaging
NMS: Neuroleptic malignant syndrome
PCR: Polymerase chain reaction
PERM: Progressive encephalomyelitis with rigidity and myoclonus
SPS: Stiff person syndrome
